# Supplementary material for: Embryo-scale epithelial buckling forms a propagating furrow that initiates gastrulation
Source: Nat Commun. 2022 Jun 10;13:3348. doi: 10.1038/s41467-022-30493-3 (PMC9187723; doi:10.1038/s41467-022-30493-3)
Supplement: Supplementary file 1 — Supplementary Information and supplementary figures [file 41467_2022_30493_MOESM1_ESM.pdf]

Supplementary Information

Embryo-scale epithelial buckling forms a propagating furrow that initiates gastrulation

Julien Fierling Alphy John Barthélémy Delorme Alexandre Torzynski
Guy B. Blanchard Claire M. Lye Anna Popkova Grégoire Malandain Bénédicte Sanson
Jocelyn Étienne Philippe Marmottant Catherine Quilliet Matteo Rauzi

Brief introduction to the strain and stress tensors

We would like to remind the reader of a few facts concerning tensors in mechanics.

Both the strain and the stress within the material need to be represented by second-order tensor fields, that is, that for each point within the material there is a local tensor associated. For a linear elastic material, the stress and strain tensors are linearly related to one another, see Eq. 2 below.

It is not in itself necessary that the variations of these tensors in space are smooth (that is, that for two given points in the material close enough, the tensors have small difference), but some smoothness arises from the mechanical balance that the stress tensor has to obey (which imposes that some derivatives of the stress tensor are small) and, as a result, for the strain tensor through Eq. 2.

An intuitive way to understand what the stress tensor $\boldsymbol{\sigma}(\boldsymbol{r})$ corresponds to is to realise that it predicts how the material would move if a cut of infinitesimal length ℓ was done in the given location $\boldsymbol{r}$. This is an idealised version of the biophysical experiment of laser ablation, where the cut length would be infinitesimal and the cut width exactly zero. For simplicity we treat it in the case of a two-dimensional material, which is the case of our model. Let us denote by the unit vector $\boldsymbol{e}$ the direction orthogonal to the laser ablation. Note of course that $-\boldsymbol{e}$ is also a unit vector orthogonal to the laser direction, pointing to the other side. To fix ideas and without loss of generality, let's define $\boldsymbol{e}$ as the right-pointing vector, and $-\boldsymbol{e}$ as the left-poiting vector. The dot product of the stress tensor with the direction vector, $\boldsymbol{f} = \boldsymbol{\sigma} \cdot \boldsymbol{e}$, is the vector of (lineic[1]) force that the material

[1] that is, the force per unit length, the total force being $\ell \boldsymbol{f}$, product of the lineic force with the length of the cut. For simplicity, we

situated to the right of the cut exerts on the line of cut, and $-\boldsymbol{f} = \boldsymbol{\sigma} \cdot (-\boldsymbol{e})$ the equal and opposite vector of force that the material situated to the left of the cut exerts on the line of cut. These equal and opposite forces pre-exist the cut: before a cut is done, the material is in mechanical balance because there is continuity of the material across the cut-line and the forces cancel. Once the cut is made, this is not the case anymore, and if one would want to maintain the material in its original shape in spite of the cut, one would have to "hold" both sides with respectively a force $-\boldsymbol{f}$ applied to the one to the right and $\boldsymbol{f}$ applied to the one on the left. If there is nothing to "hold" in place the sides of the cut, there will be motion: in a first approximation, if there is some frictional resistance to it, the initial velocity with which the sides of the cut open will be proportional to $\boldsymbol{f}$ and $-\boldsymbol{f}$ in the right and left side respectively.

The stress is said isotropic if the magnitude of the force $\boldsymbol{f}$ is the same for any cut direction $\boldsymbol{e}$. In this case, the vector $\boldsymbol{f}$ is always perfectly aligned with $\boldsymbol{e}$. If the stress is not isotropic, then for an infinitesimal cut it can be shown that there are exactly two directions $\boldsymbol{e}_1$ and $\boldsymbol{e}_2$ for which $\boldsymbol{f}_1 = \boldsymbol{\sigma} \cdot \boldsymbol{e}_1$ and $\boldsymbol{f}_2 = \boldsymbol{\sigma} \cdot \boldsymbol{e}_2$ are respectively aligned with $\boldsymbol{e}_1$ and $\boldsymbol{e}_2$. For any other direction $\boldsymbol{e}$ of the cut, the vector $\boldsymbol{f}$ will not be exactly along $\boldsymbol{e}$: the component of $\boldsymbol{f}$ orthogonal to $\boldsymbol{e}$ is called the shear force. The directions $\boldsymbol{e}_1$ and $\boldsymbol{e}_2$ are called the principal directions of the stress, they are orthogonal to one another. Since they are unit vectors, it is sufficient to give one orientation angle to define completely $\boldsymbol{e}_1$ and $\boldsymbol{e}_2$: for instance, in our case, giving the angle θ of $\boldsymbol{e}_1$ with the AP direction in the mesoderm describes the principal directions of the stress completely. It can be shown that the stress tensor can then be written as $\boldsymbol{\sigma} = \sigma_1 \boldsymbol{e}_1 \otimes \boldsymbol{e}_1 + \sigma_2 \boldsymbol{e}_2 \otimes \boldsymbol{e}_2$, where $\otimes$ is the dyadic product and σ_1, σ_2 are called the principal stresses. As a result, the stress tensor for our case of a two-dimensional material, it has three degrees of freedom, σ_1, σ_2 and θ, and it can thus be represented as an ellipse or as the cross formed by the two main axes of an ellipse on the surface of the material (see e.g. Supp. Fig. 1b) with the caveat that either or both of σ_1, σ_2 can be negative (which corresponds to compressive stress in either or both direction, whereas a positive sign denotes tensile stress). The trace of the stress tensor can then easily be expressed as $\sigma_1 + \sigma_2$, it is one of the invariants of the tensor and is convenient to represent a level of stress with a scalar (see e.g. Fig 2b).

The strain tensor is described in details in the context of morphogenesis by [1].

---

will name "force" the lineic force in what follows.

Role of viscoelastic relaxation in the presence of an exponential increase in stress

We have used a purely elastic model and have found good agreement with *in vivo* deformations and rates of deformation. This may seem in contradiction with the fact that actomyosin is a viscoelastic material [2], and that its relaxation should be apparent in the rates of deformation. In this section, we show that the context of an exponentially increasing stress explains why the material behaviour may not differ from the one of an elastic material.

Let us suppose that a material is at rest until time $t = 0$, and then submitted to an exponentially increasing stress, $\sigma = \sigma_0(e^{t/\tau} - 1)$. In an elastic material of elastic shear modulus G, the strain will be $\varepsilon_e(t) = \frac{\sigma_0}{2G}(e^{t/\tau} - 1)$. In a viscous liquid of viscosity η, the strain will be $\varepsilon_v(t) = \frac{\sigma_0}{2\eta}(\tau e^{t/\tau} - t)$. In a Maxwell viscoelastic liquid of relaxation time τ_r and viscosity η_{ve}, the strain will be $\varepsilon_{ve}(t) = \frac{\sigma_0}{2\eta_{ve}}\left((\tau + \tau_r)e^{t/\tau} - t\right)$. For $t < \tau$, the responses differ clearly, however the strains are small and thus difficult to quantify with adequate precision *in vivo*. For $t > \tau$, the constant or the linear term in each of these expressions becomes negligible compared to the exponential part, which is directly linked with the exponential load that is imposed. The difference between the asymptotic behaviours then is only in terms of a multiplicative factor, which means that the behaviours are indistinguishable in terms of strain patterns. Indeed, they are identical if material parameters are such that $G = \eta/\tau = \eta_{ve}/(\tau + \tau_r)$ and could thus be discriminated only if we could experimentally change τ without affecting the material parameters. This is difficult to achieve since MyoII, whose rate of increase is $1/\tau$, is also known to affect the elasticity and viscosity of actomyosin [3].

We thus find that while the initial transient differs, elastic, viscous and viscoelastic models will give the same patterns of strain in the case of a flow forced by exponentially increasing loading of the material.

Numerical simulations

Geometry of the elastic surface of the model

Following [4], we approximate the initial embryo shape by a closed surface of equation:

$$\Gamma_0 = \left\{ \left(\frac{x}{R_{AP}}\right)^2 + \left(\frac{y}{R_{DV}}\right)^2 + \left(\frac{z}{R_{DV}} - Z_p\left(\frac{x}{R_{AP}}\right)^2\right)^2 = 1 \right\}$$

This describes a prolate ellipsoid, with circular cross-sections. The mid-cross-section at $x = 0$ is a circle of radius R_{DV} centered at $(x, y, z) = (0, 0, 0)$. For a positive parameter Z_p, this ellipsoid is bent with the center of cross-sections offset towards increasing z as $|x|$ increases. In particular, the poles along the long axis are at the positions $(x, y, z) = (\pm R_{AP}, 0, Z_p)$.

As in [4], we choose $R_{AP} = 3R_{DV}$ and $Z_p = 0.45$, which defines the shape corresponding to the initial equilibrium configuration of the elastic surface in terms of in-plane stress. While all calculations are done in nondimensional units of R_{DV}, the dimensional counterparts of all quantities are then calculated with $R_{DV} = 90$ µm.

The elastic surface $\Gamma(\sigma_a)$ is then defined as the surface of minimal elastic energy for a given pre-stress field σ_a. The spatial dependence of σ_a is described in the main text, the corresponding rheology is described in what follows.

Additionally, two constraints are applied to the configuration of the elastic surface Γ. First, it encloses a constant volume independent of σ_a and equal to the initial volume of Γ_0. This models the fact that the permeability of the apical surface of the epithelium is low, such that water fluxes across it are assumed to be zero during the process of initial furrow formation. This gives rise to pressure forces which are dependent on σ_a and are of uniform magnitude in space. Second, Γ is itself enclosed in an undeformable vitelline membrane. The vitelline membrane is defined as a closed surface Γ_V parallel to the initial elastic surface Γ_0, at a distance $2.5 \times 10^{-3} R_{DV} \simeq 0.2$ µm towards the exterior. Non-interpenetration of the elastic surface with the vitelline membrane is simulated by Surface Evolver constraint algorithm [5], a penalty method based on a level set function ensuring that the elastic surface is in the interior side of Γ_V.

Elastic model

The deformation of an elastic layer of non-vanishing thickness can be modelled using a surface with two local additive contributions: bending and in-plane deformation [6, 7].

The bending surface energy density writes $\frac{1}{2}\kappa\,(c - c_0)^2$ where κ is the bending modulus of the surface, $c = \frac{1}{R_1} + \frac{1}{R_2}$ its mean curvature (R_i are the principal algebraic curvature radii), and c_0 the (local) intrinsic curvature, taken to be the initial one in this work [6, 8].

We now proceed to describe the part of the surface energy density due to in-plane deformation. In a

continuum description of in-plane deformations, the gradient deformation matrix $\mathbf{F}^c$, with $F^c_{ij} = \frac{\partial X_i}{\partial x_j}$ where (X_1, X_2), describes the deformed position of a material point initially at the (x_1, x_2) position (the indices stand for any two coordinates describing a surface), allow to define the right Cauchy-Green deformation tensor $(\mathbf{F}^c)^T\mathbf{F}^c$ and the Lagrangian finite strain tensor $\boldsymbol{\epsilon} = \left((\mathbf{F}^c)^T\mathbf{F}^c - \mathbf{I}\right)/2$, a widely used measure of how a material piece of surface locally differs before and after deformation.

The elasticity of the surface is described by the Hookean model, where the (locally) in-plane stress tensor $\boldsymbol{\sigma}$ is linked to the (locally) in-plane strain tensor $\boldsymbol{\epsilon}$. The model surface is considered as isotropic in plane, which allows to describe full elasticity using only two parameters, the 2D Young modulus $Y_{2\text{D}}$ and the 2D Poisson ratio $\nu_{2\text{D}}$. The surface energy density of in-plane deformation in this model can be expressed in two different useful forms:

$$\begin{aligned} e_{def} &= \frac{Y_{2\text{D}}}{2(1+\nu_{2\text{D}})}\left[\text{Tr}(\boldsymbol{\epsilon}^2) + \frac{\nu_{2\text{D}}}{1-\nu_{2\text{D}}}(\text{Tr}\boldsymbol{\epsilon})^2\right] \\ &= \frac{\chi_{2\text{D}}}{2}(\text{Tr}\boldsymbol{\epsilon})^2 + \mu_{2\text{D}}\left(\text{Tr}(\boldsymbol{\epsilon}^2) - \frac{1}{2}(\text{Tr}\boldsymbol{\epsilon})^2\right) \end{aligned} \quad (1)$$

where $\chi_{2\text{D}}$ and $\mu_{2\text{D}}$ are, respectively, the 2D compression and shear moduli:

$$\chi_{2\text{D}} = \frac{Y_{2\text{D}}}{2(1-\nu_{2\text{D}})} \qquad \mu_{2\text{D}} = \frac{Y_{2\text{D}}}{2(1+\nu_{2\text{D}})}$$

In-plane stresses write:

$$\boldsymbol{\sigma} = \chi_{2\text{D}}(\text{Tr}\boldsymbol{\epsilon})\mathbf{P} + 2\mu_{2\text{D}}\left(\boldsymbol{\epsilon} - \frac{1}{2}(\text{Tr}\boldsymbol{\epsilon})\mathbf{P}\right) \quad (2)$$

with $\mathbf{P} = \mathbf{I} - \boldsymbol{n} \otimes \boldsymbol{n}$ the projection tensor along the surface.

We nondimensionalise the parameters with $\tilde{\chi}_{2\text{D}} = \chi_{2\text{D}}R^2_{DV}/\kappa$ and $\tilde{\mu}_{2\text{D}} = \mu_{2\text{D}}R^2_{DV}/\kappa$. All figures are shown for the choices $\tilde{\chi}_{2\text{D}} = 50$ and $\nu_{2\text{D}} = 0$.

Numerical approach

We describe here the approach generally used in the Surface Evolver [5]. Although these implementation details are not specific to our approach, they are necessary to introduce the numerical technique for simulation

pre-strain in the next paragraph.

In a finite element description, $\mathbf{F}^c$ is approximated locally by $\mathbf{F}$, the matrix of the linear transformation from the unstrained to the strained facet, written in a local basis. Let $\mathbf{S} = [S_1, S_2]$ be the 2×2 matrix formed by any 2 sides of the unstrained facet ABC,

$$\mathbf{S} = \begin{bmatrix} x_B - x_A & x_C - x_A \\ y_B - y_A & y_C - y_A \end{bmatrix},$$

and $\mathbf{W}$ the corresponding matrix of the same facet in the deformed configuration. Then $\mathbf{W} = \mathbf{FS}$ which rewrites $\mathbf{F} = \mathbf{WS}^{-1}$. With $\mathbf{F}^T = \left(\mathbf{S}^{-1}\right)^T \mathbf{W}^T$, one gets the numerical implementation for approximating the Lagrangian finite strain tensor in each facet of the finite element mesh:

$$\mathbf{E} = \frac{1}{2}\left(\left(\mathbf{S}^{-1}\right)^T \mathbf{W}^T \mathbf{W} \mathbf{S}^{-1} - I\right)$$

Note that this expression is invariant with respect to permutations between A, B and C.

In practice, since $\boldsymbol{\epsilon}$ appears in Eq. 1 only through $\mathrm{Tr}\left(\boldsymbol{\epsilon}^2\right)$ and $\mathrm{Tr}\,\boldsymbol{\epsilon}$, one can use an alternative representation of the strain, namely $\mathbf{E}^* = \left(\mathbf{S}^T\right)^{-1} \mathbf{E}\mathbf{S}^T$, because due to matrix properties, $\mathrm{Tr}\,\mathbf{E}^* = \mathrm{Tr}\,\mathbf{E}$ and $\mathrm{Tr}\left(\mathbf{E}^{*2}\right) = \mathrm{Tr}\left(\mathbf{E}^2\right)$.

This definition is equivalent to $\mathbf{E}^* = \frac{1}{2}\left(\mathbf{W}^T\mathbf{W}\mathbf{S}^{-1}\left(\mathbf{S}^{-1}\right)^T - \mathbf{I}\right)$, or $\mathbf{E}^* = \frac{1}{2}\left(\mathbf{G}_W\left(\mathbf{G}_S\right)^{-1} - \mathbf{I}\right)$, where $\mathbf{G}_W = \mathbf{W}^T\mathbf{W}$ and $\mathbf{G}_S = \mathbf{S}^T\mathbf{S}$ are the (real and symmetric, hence diagonalisable) Gram matrices of, respectively, the strained and unstrained facets. Since Gram matrices, that make only intervene the square lengths and the scalar product of the two sides choosen to determine the facets, are easy to calculate from side lengths only, the alternative definition E^* is preferred in the minimisation algorithm used for the equilibrium situations [5].

Pre-strain and pre-stress

Pre-strain corresponds to a reduction of the equilibrium configuration area. In order to apply pre-strain in a given facet, we multiplied the Gram matrix of the unstrained facet by a factor $\gamma < 1$ (which amounts to multiply the dimensions of the equilibrium configuration of the facet by $\sqrt{\gamma}$):

$$\mathbf{G}_S^\gamma = \gamma \mathbf{G}_S$$

This approach can be compared to morphoelasticity, see [9].

The alternative deformation tensor hence becomes:

$$\begin{aligned}\mathbf{E}^*_\gamma &= \frac{1}{2}\left(\frac{1}{\gamma}\mathbf{G}_W\left(\mathbf{G}^\gamma_S\right)^{-1} - \mathbf{I}\right)\\ &= \frac{1}{\gamma}\mathbf{E} - \frac{\gamma-1}{2\gamma}I\end{aligned}$$

The trace is easily calculated:

$$\mathrm{Tr}(\mathbf{E}^*_\gamma) = \frac{1}{\gamma}\mathrm{Tr}\mathbf{E} - \frac{(\gamma-1)}{\gamma}$$

The stress thus becomes:

$$\begin{aligned}\sigma_\gamma &= \chi_{2D}(\mathrm{Tr}\mathbf{E}^*_\gamma)I + 2\mu_{2D}\left(\mathbf{E}^*_\gamma - \frac{1}{2}(\mathrm{Tr}\mathbf{E}^*_\gamma)\mathbf{I}\right)\\ &= \chi_{2D}\left[\frac{1}{\gamma}\mathrm{Tr}\mathbf{E} - \frac{(\gamma-1)}{\gamma}\right]\mathbf{I} + 2\mu_{2D}\left(\left[\frac{1}{\gamma}\mathbf{E} - \frac{\gamma-1}{2\gamma}\mathbf{I}\right] - \frac{1}{2}\left[\frac{1}{\gamma}\mathrm{Tr}\mathbf{E} - \frac{(\gamma-1)}{\gamma}\right]\mathbf{I}\right)\\ &= \frac{\chi_{2D}}{\gamma}\left[\mathrm{Tr}\mathbf{E} - (\gamma-1)\right]\mathbf{I} + 2\frac{\mu_{2D}}{\gamma}\left(\mathbf{E} - \frac{1}{2}(\mathrm{Tr}\mathbf{E})\mathbf{I}\right)\end{aligned}$$

Area pre-strain is thus equivalent to an isotropic pre-stress $\sigma_a = \chi_{2D}\frac{\gamma-1}{\gamma}$. Note that the apparent elastic moduli of the material are also increased by a factor $1/\gamma$, due to the fact that their equilibrium configuration is of dimensions $1/\gamma$-fold smaller than their intial configuration, with respect to which strain is calculated.

Supplementary References

1. Blanchard, G. B., Kabla, A., Schultz, N., Butler, L., Sanson, B., Gorfinkiel, N., Mahadevan, L. & Adams, R. Tissue tectonics: morphogenetic strain rates, cell shape change and intercalation. *Nature Methods* **6,** 458–464 (2009).

2. Keller, M., Tharmann, R., Dichtl, M. A., Bausch, A. R. & Sackmann, E. Slow filament dynamics and viscoelasticity in entangled and active actin networks. *Philosophical Transactions of the Royal Society of London Series a-Mathematical Physical and Engineering Sciences* **361,** 699–711 (1805 2003).

3. Murrell, M., Oakes, P. W., Lenz, M. & Gardel, M. L. Forcing cells into shape: the mechanics of actomyosin contractility. *Nat Rev Mol Cell Biol* **16,** 486–498. doi:10.1038/nrm4012 (8 2015).

4. Dicko, M., Saramito, P., Blanchard, G. B., Lye, C. M., Sanson, B. & Étienne, J. Geometry can provide long-range mechanical guidance for embryogenesis. *PLoS Comput Biol* **13,** e1005443. doi:10.1371/journal.pcbi.1005443 (3 2017).

5. Brakke, K. A. The Surface Evolver. *Experimental Mathematics* **1,** 141–165. doi:10.1080/10586458.1992.10504253 (2 1992).

6. Landau, L. D. & Lifshitz, E. F. *Theory of elasticity Course of theoretical physics* **7** (Pergamon Press, 1970).

7. Ben Amar, M. & Pomeau, Y. Crumpled paper. *Proc. R. Soc. Lond. A* **453,** 729–755. doi:10.1098/rspa.1997.0041 (1959 1997).

8. Helfrich, W. Elastic properties of lipid bilayers: Theory and possible experiments. *Z. Naturforsch.* **28C,** 693–793 (1973).

9. Goriely, A. *The Mathematics and Mechanics of Biological Growth* (Springer, 2017).

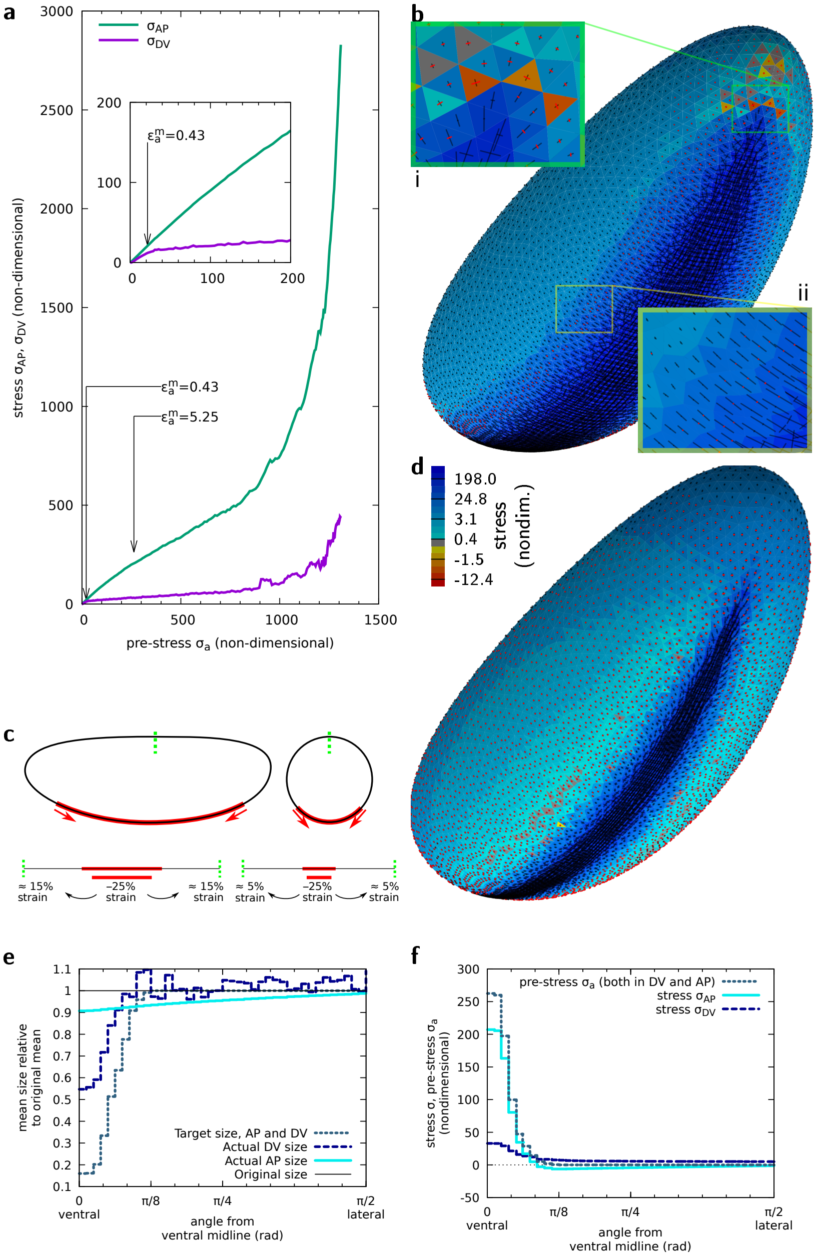

Supplementary Figure 1: Tension anisotropy increases with increased actomyosin contractility. (**a**) Evolution of AP and DV stresses at the ventral midline as a function of the pre-stress σ_a. (**b**) Principal stresses in each facet for midline pre-strain $\varepsilon_a^m = 0.43$. Black segments, positive principal stress (tensile); red segments, negative principal stress (compressive) along the corresponding directions. Facet colours, sum of the principal stresses, see **d** for colour code. Inserts (i) and (ii), 3× zoom of the regions outlined in white and in yellow. In (ii), tensor components are further enlarged by a factor 2. (**c**) Representation in sagittal and transverse sections of an example 25% strain in the ventral region and of the strain it implies in other regions if the embryo shape is unchanged. (**d**) Principal stresses in each facet for midline pre-strain $\varepsilon_a^m = 5.25$. Same colour code as **b**, nondim., nondimensional. (**e**) Same as Fig. 2a but for $\varepsilon_a^m = 5.25$. (**f**) Same as Fig. 2c but for $\varepsilon_a^m = 5.25$.

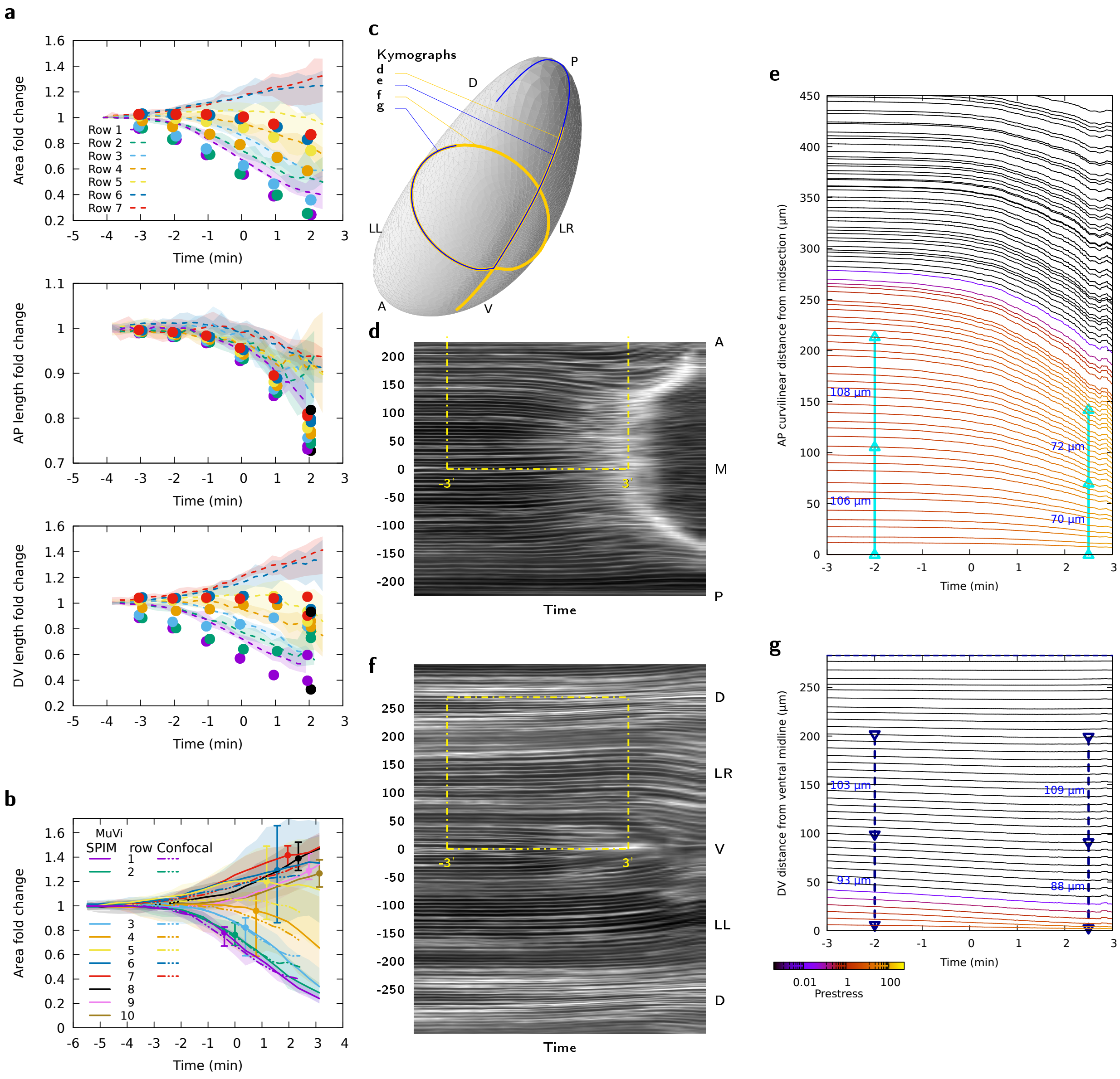

Supplementary Figure 2: Surface shape changes in vivo and in the mechanical model. (**a**) Time evolution of apical area, AP and DV sizes of cells at different distances from the midline, in confocal experiments (lines, $n = 3$ embryos, shaded area, minimum and maximum) and simulations (symbols). (**b**) Time evolution of apical area of cells in MuVi SPIM (solid lines, $n = 3$ embryos, shaded area and error bars, minimum and maximum) and confocal experiments (dash dot dot lines, $n = 3$ embryos, shaded area, minimum and maximum) (**c**) Localization of kymographs of panels **d**–**g** on the apical surface of the embryo. (**d**) Kymograph of membrane signal in MuVi SPIM along AP (mid-sagittal line). M, mid; A, anterior; P, posterior, coordinates from mid-transverse plane in μm. Yellow box delineates the zone also shown in panel **e**. (**e**) Same as **d** in simulations, with colour coded pre-stress value (see colour bar in **g**) and quantification of the decrease of length between mid-transverse point and points marked with symbols. (**f**) Kymograph of membrane signal in MuVi SPIM along DV (mid-transverse line). V, ventral; LR, lateral right; LL, lateral left; D, dorsal, coordinates from the ventral midline in μm. (**g**) Same as **f** in simulations.

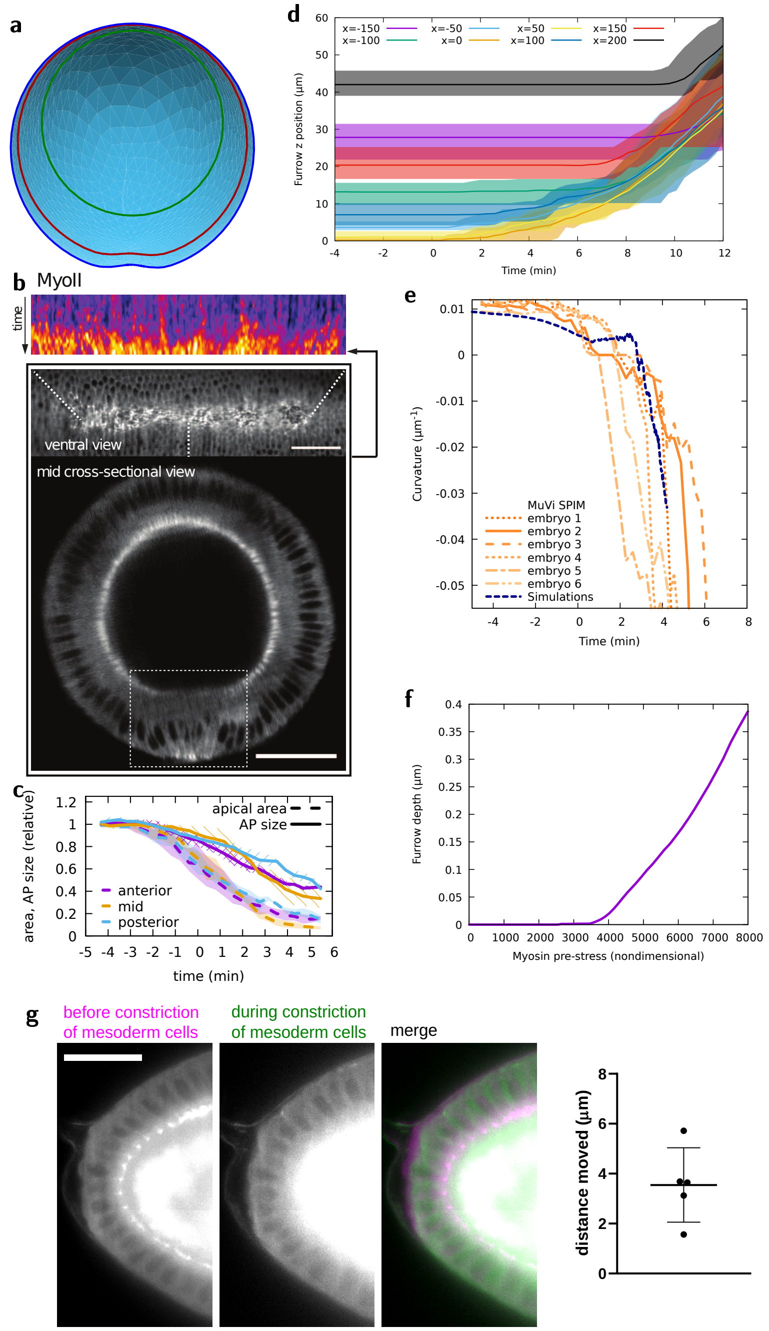

Supplementary Figure 3: Embryo shape changes during furrow propagation. (**a**) See-through view of embryo shape in simulations at $t = 4'12''$ from the posterior end. (**b**) Top, kymograph showing MyoII accumulation along the AP axis. Mid and bottom, ventral and mid-transverse view of the embryo before folding, respectively. Panel shows representative case, $n = 5$ embryos. Scale bars 50 μm. (**c**) Time evolution of apical area (dashed lines) and AP size (solid lines) of cells along the ventral midline in anterior, mid and posterior 60 μm bins. Shaded and hatched areas, min and max over $n = 3$ embryos, total number of cells $n = 87$. (**d**) Ventral furrow apex position at different AP positions. Lines represent medians and shaded areas the interval between min and max values. The analysis was performed on $n = 6$ embryos. Negative and positive x values indicate more anterior and more posterior positions, respectively. (**e**) Curvature of the ventral tissue along the DV axis as a function of time, with individual data from $n = 6$ MuVi SPIM embryos. (**f**) Depth of the ventral furrow as a function of the pre-stress σ_a applied. (**g**) Inward movement of the anterior pole during apical constriction of mesoderm cells. Magenta is before apical constriction and green is during apical constriction. The vitelline membrane is visible by auto-fluorescence. Panel shows representative case. Analyses performed on $n = 5$ embryos, data are presented as mean values +/- standard deviation. Scale bar 20 μm.
