# Supplementary material for: Embryo-scale epithelial buckling forms a propagating furrow that initiates gastrulation
Source: Nat Commun. 2022 Jun 10;13:3348. doi: 10.1038/s41467-022-30493-3 (PMC9187723; doi:10.1038/s41467-022-30493-3)
Supplement: Supplementary file 3 — Description of Additional Supplementary Files [file 41467_2022_30493_MOESM3_ESM.pdf]

Description of Additional Supplementary Files

File Name: Supplementary Movie 1
Description: Time-lapse showing actomyosin network recoil after IR fs laser ablation and eventual network recovery. Manual refocusing was performed at time point 3:41 to follow the network moving inward. Scale bar 10 µm.

File Name: Supplementary Movie 2
Description: Time-lapse showing mesoderm internalization failure as a consequence of periodic ventral tension inhibition. The ventral actomyosin network is periodically ablated using a IR fs laser over a grid patterned ROI. Scale bar 10 µm.

File Name: Supplementary Movie 3
Description: 3D rendering of segmented apical surfaces of ventral cells. Scale bar 50 µm.

File Name: Supplementary Movie 4
Description: Time-lapse showing digital sections along the sagittal plane (top) and along different cross-section planes at different AP positions in a wild-type embryo. Scale bar 100 µm.

File Name: Supplementary Movie 5
Description: Time-lapse showing different cross-section planes at different AP positions in a wild-type embryo and eye guide highlighting furrow apex position. Scale bar 100 µm.

File Name: Supplementary Movie 6
Description: Time-lapse showing digital sections along the sagittal plane (top) and along different cross-section planes at different AP positions in a slam-dunk- embryo. Scale bar 100 µm.

File Name: Supplementary Movie 7
Description: Time-lapse showing ventral tissue flattening along a line connecting cauterised loci. Scale bar 100 µm.
